# Resource stewardship learning needs in undergraduate medical training: a cross-sectional study

**DOI:** 10.12688/mep.20877.1

**Published:** 2025-07-28

**Authors:** David Houle, Anton Volniansky, Sophie Rodrigues-Coutlée, Merve Kulbay, René Wittmer

**Affiliations:** 1 Medecine, Universite de Montreal, Montreal, Québec, Canada; 2 Department of Ophthalmology & visual sciences, McGill University, Montreal, Québec, Canada; 3 Department of family medicine and emergency medicine, Universite de Montreal, Montreal, Québec, Canada

**Keywords:** Medical students, medical education, resource stewardship, Choosing Wisely

## Abstract

**Background:**

No clear guidelines for Canadian medical schools regarding the integration of resource stewardship (RS) in the training of tomorrow’s physicians exist. This study aimed to determine the learning needs of undergraduate medical students on RS in Quebec.

**Methods:**

An online survey was distributed to all medical students in Quebec from January 12 ^th^ to April 27 ^th^, 2021. A cross-sectional study was then performed. In total, 900 medical students (23.2% of all Quebec medical students) were included in this study.

**Results:**

RS teaching was perceived as being insufficient. Despite 69% of students knowing the existence of Choosing Wisely, only 21.7% considered RS teaching to sufficiently develop their critical thinking. While students claimed that RS's current teaching methods are predominantly theoretical, a preference for more practical learning approaches was noted. Approximately 71% of students undergoing clerkship rotations acknowledged the significance of role models in fostering critical thinking skills related to RS. Heterogeneity was noted across medical schools.

**Conclusions:**

This study confirms that according to students’ perspective, RS is important yet insufficiently and heterogeneously taught in Quebec. Establishing guidelines regarding RS teaching in medical schools could provide equal learning opportunities. Furthermore, encouraging role models to be part of this teaching effort may also strengthen student’s critical thinking regarding RS.

## Introduction

Health care systems have been identified as a great contributor to increased greenhouse gas (GHG) emissions ^1^. In the United States, in 2013, it was projected that GHG emissions associated with health care would cause up to nearly 320 000 disability-adjusted life-years in health damages ^1^. The Canadian health care system was established to be responsible for nearly 5% of the country’s total GHG emissions, translating to 23 000 disability-adjusted life-years, making it the worse worldwide in terms of greenhouse pollution ^2, 3^. Leaders around the world have recognized the importance to decrease the negative impact of health care systems on the environment ^4– 7^, and therefore numerous actions have been proposed to improve sustainability in this field ^8– 11^.

Sustainability in health care is impacted when clinical practices are i) effective but underused, ii) ineffective and overused, or iii) misused ^12, 13^. In the Canadian health care system, the proportion of tests, treatments, and procedures that are potentially unnecessary was estimated to reach up to 30% ^12^. Fostering resource stewardship (RS) among physicians is fundamental since the majority of health care costs was estimated to stem from doctors' decisions ^14^ and quality of care depends on effective resource allocation ^15^. Three fields of actions for physicians have been proposed to incentivise value in health care, which consists of education, mentorship, and audit and feedback ^16^. Health care organizations should i) educate physicians regarding quality of care and decision making through easy access to educational resources and programs, ii) promote reflective practice, and iii) establish tools for individual and group performance measurements and management ^16^.

Choosing Wisely Canada (CWC) campaign was created in 2014 in the efforts to minimize unnecessary intervention in the health care system ^17^. Implementation of Choosing Wisely recommendations can lead to the reduction of low-value care ^18– 20^. Furthermore, engaging learners in RS has been identified as a potential way to foster better use of healthcare resources ^21, 22^. Despite the need for high-value care, the integration of RS in the medical training is slow to be implemented both during medical school and residency ^23– 25^. The Royal College of Physicians and Surgeons of Canada recognized the importance of RS through its integration into the CanMEDS framework ^26^, but there are no clear guidelines for Canadian medical schools regarding the integration of RS in the training of tomorrow’s physicians. Up until now, few studies have analysed the student perspective on RS education during undergraduate medical studies in the United States and in Canada. To our knowledge, the learning needs in terms of RS remains unknown in Quebec. Therefore, the aim of this study was to determine undergraduate medical students’ learning needs from students’ perspective.

## Methods

### Study type

This cross-sectional study was conducted in Quebec in Canada from January 12 ^th^ to April 27 ^th^, 2021. An official exempt letter was obtained from the Ethics Board of the Université de Montréal, namely the *Comité d’éthique de la recherche en sciences et en santé* (CERSES). This project was performed exclusively for evaluation and improvement purposes. This manuscript uses anonymous data for research and falls under the Tri-Council Policy Statement (TCPS2) article 2.4. Regarding consent, participation was entirely voluntary. Respondents could exit the survey at any time, and data was recorded only upon submission. No identifying information was collected. Participants were informed that the data would be used for educational purposes, including improving learning opportunities and producing a publicly available report. Submission of the survey was considered as implied consent. The ethics board confirmed that explicit consent was not required, as the project qualified as a quality improvement and practice evaluation initiative.

### Study population

This study involved undergraduate medical students enrolled in one of the four medical schools in Quebec (Canada) in 2021, namely Université de Montréal (UdeM), Université Laval (UL), Université de Sherbrooke (UdeS), and McGill University (McGill). Undergraduate medical students consisted of first year medical students (MS1), second year medical students (MS2), and first- and second-year clerkship students (CS). Postgraduate medical students, as well as medicine preparatory program (Med-P) students ^27, 28^ were excluded from this study. Incoherence between medical affiliation and year of study was considered as an exclusion criterion. The number of students enrolled in each year and in each medical school was obtained directly from the four medical school students’ associations.

### Study design and distribution

A bilingual (English and French) survey was created using the online platform Microsoft Form ( https://forms.office.com). The survey consisted of eleven closed-ended questions and respondents had the option of filling out an open-ended comments section to add any clarifications deemed relevant. The questions were designed with the help of an expert medical educator (R.W.) to ensure comprehensibility and coherence. The survey investigated five RS key areas: (i) awareness level, (ii) student perception of RS teaching, (iii) education methods (actual versus desired), (iv) clerkship students’ perceptions of role models’ impact on the development of critical thinking, and (v) perception of the importance of RS in future practice. A preliminary survey was administered to a focus group consisting of 12 medical students and a second expert medical educator. Comments and suggestions received were used to refine the questionnaire.

The survey was distributed to all undergraduate medical students across the four medical schools. Distribution methods included: (i) distribution lists of medical school students’ associations, (ii) online medical student courses and events, and (iii) social media exclusively accessible to medical students. As mentioned previously, data were collected anonymously and, apart from student university affiliation and level of training, no other demographic data were collected. Individuals could pause or quit at any time, and responses were only saved upon survey submission. Once the survey completed, participants were given a second link allowing them to register for a prize draw. Five (5) one-year Complete Anatomy licenses (a value of $50 each granted by 3D4Medical from Elsevier) and five (5) gift cards totaling $500 in prizes, granted by Uniforme Direct, were given at random among survey participants to promote participation in the survey.

### Data post-processing

At UL, students can complete their preclinical years in either two, two and a half, or three years. Respondents from UL in their second to third year of preclinical studies were considered as MS2. Furthermore, given that hospital settings and order of clinical rotations vary among clerkship students, first- and second-year clerkship students were combined into a single category (CS).

### Statistical analysis

An independent statistician from Centre hospitalier universitaire Sainte-Justine performed the statistical analyses using SAS statistical software (version 9.4) and the Proc Surveyfreq procedure. A weighted class adjustment was performed to compensate for the heterogeneous response rates between and within universities and to obtain estimates that are representative of the undergraduate medical students’ population in Quebec. University affiliation and number of students per year were used to define the weighted classes. Finally, to compare groups, we performed two-sample proportion test (Z test) with Python version 3.11.0 (Python Software Foundation) using Statsmodels library version 0.14.1.

## Results

**Figure 1.**
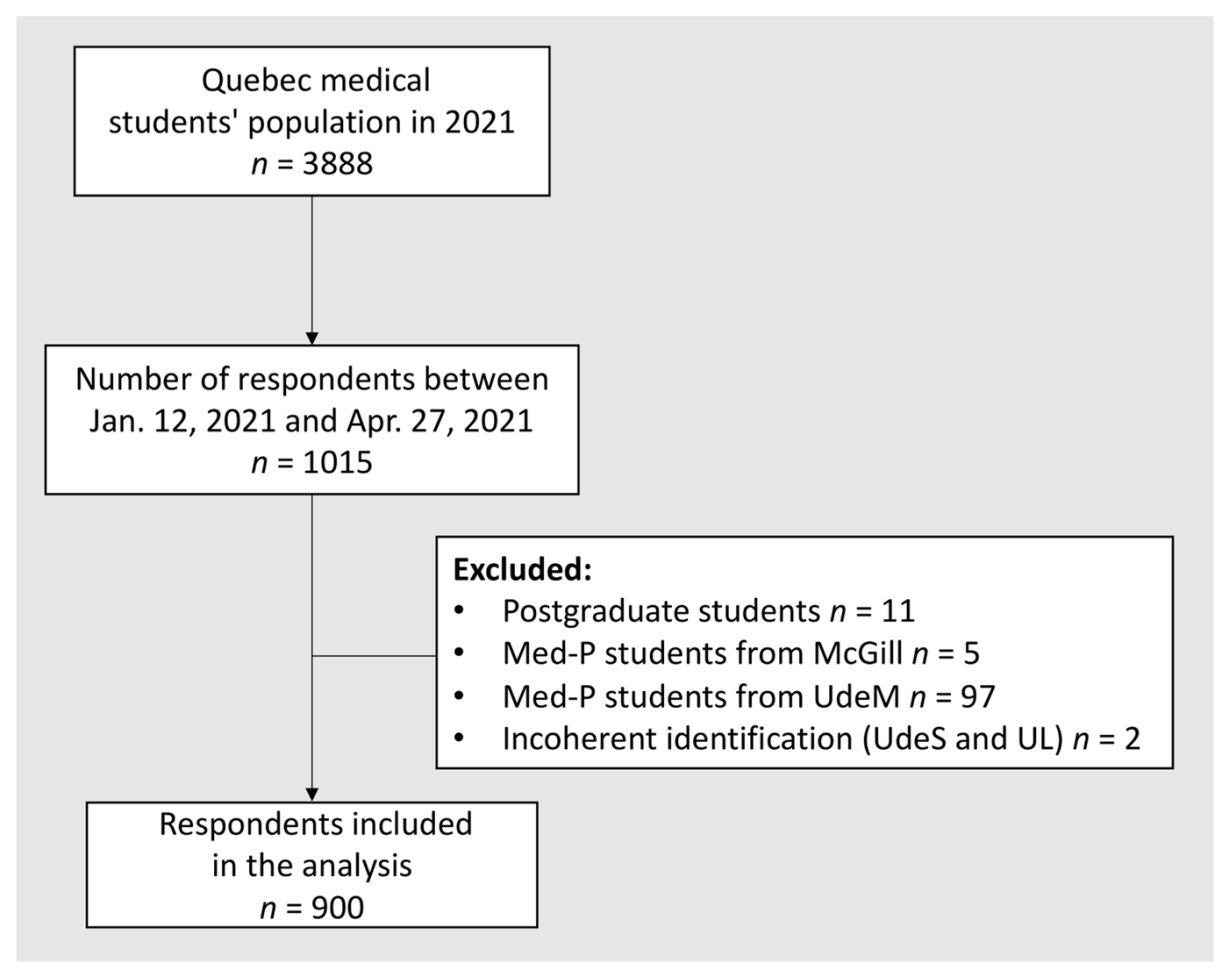
Flowchart of undergraduate medical students’ selection. Abbreviations: UdeM, Université de Montréal; UL, Université Laval; UdeS, Université de Sherbrooke; McGill, McGill University.

From January 12 ^th^ to April 27 ^th^, 2021, 1,015 respondents completed the survey. After applying exclusion criteria, 900 medical students were included in the analysis, which represents a response rate of 23.1% ( Figure 1). The distribution of participants, as well as the proportion used for weighted class adjustment are summarized in Table 1. Survey results calculated after weighted class adjustment for the undergraduate medical students’ global population are listed in Table 2. Survey results prior to weighted class adjustment can be found in **Extended data** **Table 1.**

**Table 1.** Respondent distribution among undergraduate medical students used for weighting class adjustment.

Level of Study	No. of participants (% used for weighting class adjustment) *				
	UdeM	UL	UdeS	McGill	Total
**MS1**	176 (58.7%)	71 (28.6%)	55 (24.9%)	81 (38.9%)	384 (39.3%)
**MS2**	69 (23.9%)	126 (30.6%)	61 (31.6%)	33 (15.8)	289 (26.2%)
**CS**	30 (5.3%)	59 (13.0%)	89 (21.3%)	50 (13.6%)	228 (12.6%)
**Total**	275 (23.7%)	256 (23.0%)	205 (24.6%)	164 (20.9%)	900 (23.2%)

*Numbers correspond to the number of respondents obtained for the category. Percentages correspond to the proportion of respondents obtained for the specified university and year of study. For example, in UdeM, for MS1, 176 students (among 300) filled the survey. This corresponds to a participation rate of 58,7% which was used for weighting class adjustment. Abbreviations: CS, Clerkship students (first and second year); MS1, First year medical students; MS2, Second year medical students; UdeM, Université de Montréal; UL, Université Laval; UdeS, Université de Sherbrooke; McGill, McGill University.

**Table 2.** Survey results after weighting class adjustment for the Quebec medical student’s global population.

Key area and questions	UdeM ( *Ajusted N* = 1158) %	UL ( *Ajusted* *N*= 1113) %	UdeS ( *Ajusted* *N* = 832) %	McGill ( *Ajusted* *N* = 784) %	All ( *Ajusted* *N* = 3888) %
**(i) RS awareness level**					
Are you aware of the existence of Choosing Wisely?					
Yes	82.5	57.7	62.5	68.4	68.5
No	17.5	42.3	37.5	31.6	31.5
When in your training were you first exposed to resource stewardship (appropriate use of health examinations and treatments)?					
MS1 or before	71.9	69.2	84.6	80.7	75.3
MS2	16.2	19.9	11.0	10.1	15.0
CS	4.9	6.2	4.4	4.7	5.2
Not exposed	7.0	4.7	0.0	4.6	4.5
**(ii) Student perception of RS teaching**					
Do you consider that the principles of the judicious use of health examinations and treatments are taught in the MANDATORY educational activities offered by your medical school?					
Sufficiently	17.4	20.4	55.9	27.2	27.5
Yes, but not sufficiently	70.0	72.5	41.5	66.7	64.8
No	12.6	7.1	2.6	6.1	7.8
Do you feel that the academic training you have had to date sufficiently develops your critical thinking skills with respect to the use of unnecessary health tests and treatments?					
Sufficiently	12.8	18.4	41.5	21.7	21.7
Yes, but not sufficiently	60.9	66.1	53.6	67.3	62.4
No	26.3	15.5	4.9	11.0	15.9
Based on the scale below, how much do you agree with the following statement: Would you like your faculty to introduce more content about unnecessary health tests and treatments into the medical curriculum?					
Strongly agree	52.1	41.1	28.3	32.9	40.4
Agree	40.7	47.8	51.6	53	47.4
Neutral	6.4	9.3	18.2	11.5	10.5
Disagree	0.14	1.5	1.9	2.6	1.4
Strongly disagree	0.7	0.3	0.0	0.0	0.3
**(iii) Methods of teaching**					
Since the beginning of your medical studies, under what form(s) have you been presented with content related to the judicious use of health examinations and treatments					
Lectures	73.9	58.2	20.6	80.3	60.7
PBL	59.2	45.3	90.9	44.5	57.9
OSCE	3.4	13.5	24.6	12.0	12.1
Preclinical internship	17.7	8.1	19.7	17.4	15.1
Clerkship rotation	19.8	38.1	35.9	36.5	31.7
Student initiatives	65.2	46.7	45.3	56.3	54.2
In what format would you prefer this teaching to take place?					
Lectures	73.1	65.6	53.4	56.1	63.6
PBL	57.9	62.7	59.7	68.8	61.9
OSCE	20.9	26.1	23.6	41.4	27.2
Preclinical internship	44.0	39.6	31.0	42.5	40.0
Clerkship rotation	41.9	39.8	29.0	45.2	39.6
Not a relevant topic	0.1	0.3	1.5	1.3	0.65
**(iv) Role models**					
How much do you agree with the following statement: During clerkship or residency, my supervisors and role models (supervisor, fellow, other health professionals) helped me develop my ability to choose appropriate tests and treatments for my patients? *					
Strongly agree	0.0	23.7	18.0	16.0	13.2
Agree	50	67.8	58.4	56.0	57.6
Neutral	43.3	6.8	19.1	24.0	24.8
Disagree	3.3	1.6	4.5	4.0	3.3
Strongly disagree	3.3	0.0	0.0	0.0	1.1
**(v) RS in future practice**					
Do you feel that choosing medical tests and treatments wisely will be a key issue in your future practice?					
Strongly agree	60.4	64.9	59.1	48.7	59.0
Agree	32.6	26.4	32.8	40.0	32.4
Neutral	6.5	6.5	3.8	10.5	6.8
Disagree	0.1	0.3	3.8	0.8	1.0
Strongly disagree	0.3	1.9	0.6	0.0	0.8

*Question addressed to clerkship students only. Abbreviations: CS, Clerkship students (first and second year); CWC, Choosing Wisely Campaign; RS, resource stewardship; McGill, McGill University; OSCE, objective structured clinical examination; PBL, problem-based learning; UdeM, Université de Montréal; UdeS, Université de Sherbrooke; UL, Université Laval.

### 3.1 Awareness level

**Figure 2.**
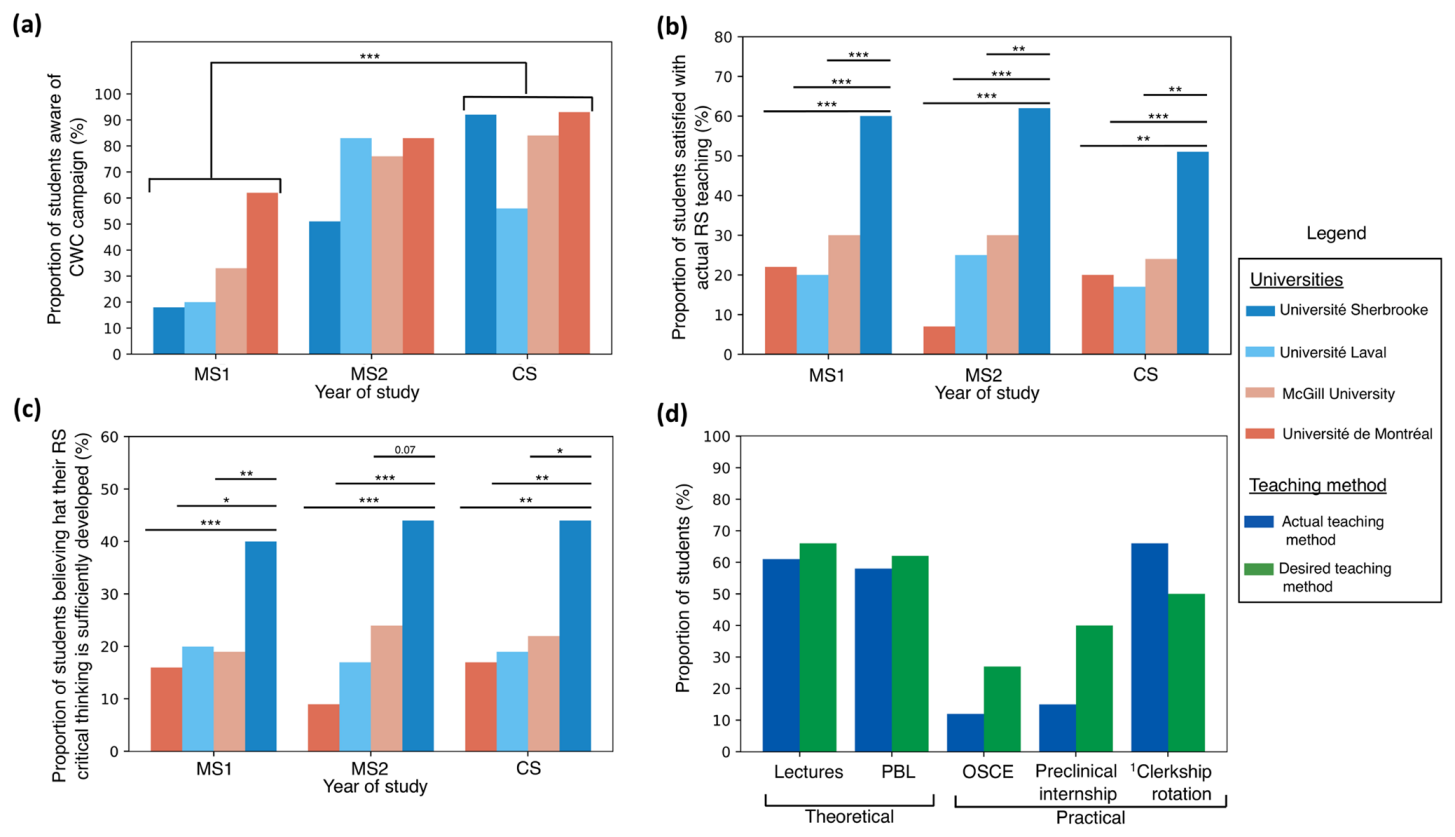
Undergraduate medical students’ ( **a**) awareness proportion of the Choosing Wisely Canada, ( **b**) satisfaction with resource stewardship teaching in mandatory academic activities, ( **c**) perception of their critical thinking and ( **d**) comparison of actual methods of resource stewards teaching to desired methods of teaching. Abbreviations: CS, Clerkship students (first and second year); CWC, Choosing Wisely Canada; MS1, First year medical students; MS2, Second year medical students; OSCE, objective structured clinical examination; PBL, problem-based learning; RS, resource stewardship; *, *p* ≤ 0.05; **, *p* ≤ 0.01; *** *p* ≤ 0.001. ^1^Only clerkship students were considered for this category.

The proportion of students that were aware of Choosing Wisely Canada (CWC) increased with medical education levels in all medical schools except UL ( Figure 2.a). Medical students from the CS group were those who were the most acquainted with the CWC campaign (global population: 68.5 %; CS: 81,4%). When comparing MS1 to CS respondents, the awareness level was significantly different ( *p* < 0.001). Furthermore, 75.3% of medical students were acquainted with RS before or during the first year of their medical training.

### Student perception of RS teaching

Less than the third of undergraduate medical students (27.5%) considered that RS was sufficiently taught in mandatory academic activities, and there was not significant difference between MS1 and CS respondents ( *p* = 0.37). However, among universities, UdeS consistently stood out in terms of satisfaction rates, with more than 50% of students considering that the teaching was sufficient ( Figure 2.b). When comparing each university to UdeS for each year of study, all the comparisons were significant ( *p* < 0.003), suggesting a greater satisfaction with RS teaching at UdeS in all levels of training.

Less than the quarter of undergraduate medical students (21.7%) considered that their academic training sufficiently developed their critical thinking concerning RS ( Figure 2.c). A similar pattern with UdeS was observed: a greater proportion of UdeS undergraduate medical students considered that their critical thinking concerning RS was sufficiently developed. Once again, when comparing each university to UdeS for each year, all the comparisons were significant (p < 0.02), except when comparing with McGill for MS2 ( *p* = 0.07). Lastly, most undergraduate medical students (87.8%) were in favor of introducing more educational content related to unnecessary tests and treatments into the academic curriculum, and there was not significant difference between MS1 and CS respondents ( *p* = 0.61).

### Teaching methods

Students reported that RS was mainly taught through theoretical methods (lecture-based classes: 60.7%; problem-based learning: 57.9%) ( Figure 2.d). It was taught less during practical activities (preclinical internship: 15.1%; objective structured clinical examination [OSCE]: 12.1%). It is in the practical area that the gap between actual and preferred education is the widest (desired preclinical internships: 40.0%; desired OSCE: 27.2%), and the trend is consistent throughout the years. Around 66.4% of CS students reported that RS was taught in clinical settings. Although more than 90% of CS students indicated that they wished for more education related to RS, only 49.6 % of them considered that this material should be taught during clerkship rotations, which is less than that is currently taught.

### Role models and importance of RS in future practice

Among CS students, 70.8% agreed that role models (e.g. supervisors, fellows, and other health professionals) have an important impact in the development of their critical thinking regarding RS. Finally, undergraduate medical students globally felt (91.4%) that RS will be a key issue in their future practice, and there was not significant difference between MS1 and CS respondents (p = 0.31).

## Discussion

This cross-sectional study assessed undergraduate medical students’ RS learning needs in Quebec, Canada. We found that although most medical students claimed to have been introduced to RS and the CWC campaign in their first year of training, few believe that it was sufficient. Few also considered that their academic training sufficiently developed their critical thinking, and most students wanted to receive additional training on RS. We also found that current teaching methods are mostly theoretical with lectures and problem-based learning prevailing, and students desire to improve the practical opportunities.

Low satisfaction rates have been observed elsewhere in Canada and the United States. Leon-Carlyle *et al.* found that over 80% of students at the University of Toronto and at Harvard felt that they had received too little formal teaching on cost-conscious decision-making and 85% would like more formal teaching on that topic ^29^. Huo *et al.* found that integration of the Choosing Wisely campaign into pre-clerkship and clerkship curricula in Canada was sufficient according to 47.0% and 63.5% students, respectively ^30^. Also, internal medicine clerkship directors in the United States and in Canada have recognized that in their opinion, RS is insufficiently taught ^31^.

Heterogeneity among universities was observed in this study in key areas (i) awareness level, (ii) satisfaction rates regarding education, and (iv) role model. Although specific reasons for the heterogeneity have not been formally investigated, a potential explanation is the absence of guidelines for medical schools regarding the integration of RS. A possible avenue to foster equal learning opportunities for medical students is the integration of RS into guidelines, for example the accreditation standards of the Committee on Accreditation of Canadian Medical Schools ^32^. Our study shows that UdeS stands out in terms of satisfaction and awareness level regarding RS. An informal discussion with colleagues from this university revealed that when students evaluate each professor and supervisors, they are asked whether their role model is promoting and applying RS to his practice. This may be an effective initiative for both role models and students to discuss RS.

As mentioned previously, students expressed a preference for maintaining the current level of theoretical teaching methods, with improving the practical opportunities, despite CS manifesting less interest into more clerkship rotation education on RS. Benbassat suggested that medical students’ observations of behaviors, specifically those of their role models, affect learning more than formal teaching ^33^. Physicians trained in high-spending regions tend to have a higher spending mean compared to those trained in low-spending regions ^34^. Residents who prescribe many tests or treatments sometimes do so because they perceive such expectations from supervisors ^35^. Huo *et al.* identified that common barriers observed in Canadian academic hospitals are (i) student’s assumption that their physician mentor know more than them (86.4%), (ii) student’s apprehension over evaluations (66.0%), and (iii) preoccupation regarding their reputation (31.2%) ^30^. We confirmed that supervisors are role models that have an important impact on the development of CS’s critical thinking regarding RS. Hence, it is important to increase awareness among supervisors as well, given their pivotal role in student guidance and leadership during clerkship rotations, which account for a significant portion of a medical student's training ^36^. The literature contains both suggestions for physicians ^37– 39^ and medical program leaders regarding the teaching of RS to trainees ^35^.

Finally, highlighting the need to prepare future physicians, we found that students highly identified RS as a key issue in their future practice. We believe ensuring that all medical students receive training on resource stewardship throughout medical training may potentially help future physicians order tests when most appropriate and integrate patient preferences wisely. Previous studies have shown that internists who trained in hospitals with lower-intensity medical practice management are more likely to de-escalate care when appropriate ^40^.

This study has limitations. First, it is unclear how our respondents might differ from non-respondents. Second, the response rates varied from year-university (minimal response rate observed is 5% for CS at UdeM). We partially minimized this component of the bias with a weighted class analysis. However, as no demographic data (e.g. age, sex, previous degree completed, etc.) was collected, it cannot be confirmed whether the characteristics of the sample correspond to the reference population. Considering that CS were generally under-represented compared to MS1 and MS2, future studies should explore alternative recruitment methods to reach CS.

In conclusion, this study highlights that according to Quebec undergraduate medical student’s perception, RS is not sufficiently taught. Students are favorable to further integration of RS in the curriculum, with both theoretical and practical activities. Since there are no current requirements regarding RS integration and discrepancies between universities were found, establishing guidelines regarding RS teaching in medical schools may be the solution to ensure equal learning opportunities. Finally, role models are also encouraged to take part in this teaching effort to help strengthen students’ critical thinking regarding RS.

## Ethic and consent

An official exempt letter was obtained from the Ethics Board of the Université de Montréal, namely the *Comité d’éthique de la recherche en sciences et en santé* (CERSES). This manuscript uses anonymous data for research and falls under the Tri-Council Policy Statement (TCPS2) article 2.4. This project was performed exclusively for evaluation and improvement purposes.

## Abbreviations

CS                Clerkship students (first and second year)

CWC            Choosing Wisely Canada

GHG            Greenhouse gas

Mcgill          McGill University

MS1             First year medical students

MS2             Second year medical students

OSCE           Objective structured clinical examination

PBL              Problem-based learning

RS                Resource stewardship

UdeM           Université de Montréal

UdeS            Université de Sherbrooke

UL                Université Laval

## Data Availability

All data related to this manuscript is publicly available. Figshare: Resource stewardship learning needs in undergraduate medical training: a cross-sectional study. The project contains the following underlying data: a dataset including respondents’ answers to the survey (Project dataset; RS_Dataset_12022025.xlsx; https://doi.org/10.6084/m9.figshare.28424417) ^41^ Figshare: Resource stewardship learning needs in undergraduate medical training: a cross-sectional study. This project contains the following extended data: survey results for the Quebec medical students by universities prior to class weight adjustment. (Extended data Table 1; Extended Data.docx; https://doi.org/10.6084/m9.figshare.28904540) ^42^ Data are available under terms of Creative Commons Attribution 4.0 International (CC BY 4.0)
