# Supplementary material for: Resource stewardship learning needs in undergraduate medical training: a cross-sectional study
Source: MedEdPublish (2016). 2025 Jul 28;15:39. [Version 1] doi: 10.12688/mep.20877.1 (PMC12982998; doi:10.12688/mep.20877.1)
Supplement: Peer review history

# Reviewer report: Reviewer response for version 1

**DOI:** 10.21956/mep.22356.r45391

**Published:** 2026-03-12

**Authors:** Julie A. Hunt

**Affiliations:** 1 Lincoln Memorial University College of Veterinary Medicine, Harrogate, USA

This paper describes a multi-institution survey of Canadian medical students at various stages of training. It investigates students' perception of their resource stewardship (RS) learning. The robust response rate is a strength of this study, as is the importance and relevance of the topic. The study analyzed the data appropriately, and data is presented in a reader-friendly format.

 The major limitation of the study, which is not mentioned by the authors, is that this is a survey about student perception of their learning. The study does not do any testing or clinical observation to find out how students behave, and it does not ask educators to report their teaching methods and strategies or what they perceive to be important in RS learning at different stages. Therefore, I think the conclusion of the study can only be that students perceived that they were insufficiently taught RS, not that they were insufficiently taught.

 The paper is Canada-centric, with the strength of being multi-institutional. It would have been helpful to describe how other countries value RS to attempt to make the paper more relevant to non-Canadian readers. I also would've liked a more robust description of what educators at the best-performing school were doing differently. Was it only the student feedback survey question about RS that caused it? Were their curricula similar?

 I'd encourage the authors to consider revising their study to address some of the comments listed above, but as is the paper does a good job of reporting the results of their survey and its applicability to medical education in Canada.

Have any limitations of the research been acknowledged?

Partly

Is the work clearly and accurately presented and does it cite the current literature?

Yes

If applicable, is the statistical analysis and its interpretation appropriate?

Yes

Are all the source data underlying the results available to ensure full reproducibility?

Yes

Is the study design appropriate and is the work technically sound?

Yes

Are the conclusions drawn adequately supported by the results?

Partly

Are sufficient details of methods and analysis provided to allow replication by others?

Yes

Reviewer Expertise:

curriculum, assessment, clinical skills simulation, educational research

I confirm that I have read this submission and believe that I have an appropriate level of expertise to confirm that it is of an acceptable scientific standard.

# Reviewer report: Reviewer response for version 1

**DOI:** 10.21956/mep.22356.r45392

**Published:** 2026-03-05

**Authors:** Devi Prasad Mohapatra

**Affiliations:** 1 Jawaharlal Institute of Postgraduate Medical Education & Research (JIPMER), Puducherry, India

The present manuscript is well written. The introduction highlights the issues of Greenhouse Gas Emissions and how it is directly contributed from the healthcare system. It also mentions the need of fostering resource stewardship among physicians as majority of healthcare costs are from doctors' decisions. This study is a cross-sectional survey among undergraduate medical students of Quebec, Canada, and has received consent exemption from the Institute Ethics Board. The study methodology is appropriate with adequate information on data post-processing as well as statistical analysis. The results are on expected lines with perceived availability of resource-stewardship learning in early undergraduate curriculum and mostly theoretical. The authors clearly mention the need of practical opportunities during undergraduate training as well as the need to improve awareness among supervisors who would guide students during their clerkships.

 The authors have acknowledged the limitations of the study; however, it is important to note that the data were collected between January and April 2021, nearly five years prior to the present submission. Given the evolving nature of undergraduate medical curricula, the authors may consider discussing any curricular updates or national initiatives related to resource stewardship that have occurred since then. This would help the readers interested to know recent changes. The authors may consider undertaking a follow-up study to evaluate how resource stewardship education and learner perceptions may have evolved over time.

Have any limitations of the research been acknowledged?

Partly

Is the work clearly and accurately presented and does it cite the current literature?

Yes

If applicable, is the statistical analysis and its interpretation appropriate?

Yes

Are all the source data underlying the results available to ensure full reproducibility?

Yes

Is the study design appropriate and is the work technically sound?

Yes

Are the conclusions drawn adequately supported by the results?

Yes

Are sufficient details of methods and analysis provided to allow replication by others?

Yes

Reviewer Expertise:

Surgical education, Competency-based medical education (CBME), Continuing professional development (CPD), Clinical reasoning and decision-making, AI in medical education, Interprofessional education.

I confirm that I have read this submission and believe that I have an appropriate level of expertise to confirm that it is of an acceptable scientific standard.
